# Turkish translation and cross-cultural adaptation of the Sport Concussion Assessment Tool 6th edition (SCAT6)

**DOI:** 10.1186/s13102-026-01784-9

**Published:** 2026-06-03

**Authors:** Fuat Yüksel, Elif Aygün Polat, Seyda Yilmaz Özal

**Affiliations:** 1 Faculty of Health Sciences, Department of Physiotherapy and Rehabilitation, Ordu University, No: 218, Altınordu, Ordu, 52220 Türkiye; 2 Faculty of Health Sciences, Department of Physiotherapy and Rehabilitation, Ankara Medipol University, Ankara, Türkiye

**Keywords:** Sports medicine, Concussion evaluation, Construct validity

## Abstract

**Background:**

Sport-related concussion are a significant public health issue. Although international criteria are available, there is no updated Turkish version of Sport Concussion Assessment Tool 6 (SCAT6). This research was performed to translate and cross-culturally adapt SCAT6 and instructions into Turkish (SKDA6™).

**Methods:**

The 8-step translation and adaptation process was based on international guidelines. Lawsha method was used to measure the content validity by 10 experts. Reliability was determined in 27 athletes using intra-class correlation coefficients and inter-rater assessments, conducted at 1 and 2 weeks following initial testing. The relationship between the SKDA6™’s ‘Symptom Evaluation’ section, completed by the athlete, and the Oslo Sports Trauma Research Centre Questionnaire on Health Problems (OSTRC-H) total score was assessed using Spearman’s correlation coefficients.

**Results:**

The ultimate Content Validity Index was 0.900. The severity of symptoms was significantly correlated to OSTRC-H questionnaire (*r* = 0.666, *p* < 0.001). Good inter-rater reliability was found for assessment of symptoms by SKDA6™ (ICC = 0.902) and moderate-good within cognitive and balance subsections.

**Conclusions:**

SKDA6™ is the Turkish version of the original instrument and adequately adapted culturally, while construct validity and reliability testing are conducted among Turkish-speakers in this study. This study has addressed a significant gap in the field by translating a standard concussion assessment tool for use by healthcare professionals in Türkiye.

**Clinical trial number:**

NCT06739785 /13.12.2024

**Supplementary Information:**

The online version contains supplementary material available at 10.1186/s13102-026-01784-9.

## Background

Sport-related concussions (SRCs) have emerged as a significant public health concern, particularly in contact and collision sports. SRC is a traumatic brain injury caused by a direct blow to the head, neck, or body resulting in an impulsive force transmitted to the brain, leading to a range of clinical symptoms and signs that may or may not involve loss of consciousness [1]. The transient nature of clinical symptoms, ranging from cognitive and emotional disturbances to physical impairments, underscores the need for accurate diagnosis, timely management, and effective prevention strategies [2].

Global epidemiological evidence suggests that sport-related traumatic brain injury accounts for up to one-third of traumatic brain injuries in some population-based studies, with the highest incidence observed in adolescents and young adults [3]. Although there are no epidemiological data on SRCs in Türkiye, the limited data available suggest that head injuries mostly occur in young adults and are caused by falls [4].

The Sport Concussion Assessment Tool (SCAT) was developed to be used as an evaluation tool for health professionals to use in the assessment of SRCs. To date, 5 different versions of SCAT the first of which was created in 2005, have been published [5]. The latest version, SCAT6, was created at the 6th International Conference on Concussion in Sport in Amsterdam in 2022 and was published in the British Journal of Sports Medicine in 2023 [6].

In recent years, adaptations of the SCAT in different languages have been published. The existing literature includes Italian, Korean, Persian, Arabic and Chinese versions [7–11]. Additionally, Danish, German, Finnish, French, Greek and Spanish versions of SCAT6 are available on the Concussion in Sport Group (CISG) website as of April 2026 [12]. Prior to Sport Concussion Assessment Tool 6th Edition Turkish Version (SKDA6™,) none of them had been translated into Turkish or culturally adapted.

The lack of Turkish translation and cultural adaptation of any version of SCAT is a major deficiency. There are 16 million athletes in sport in Turkey alone. Considering the recreational athletes and Turkish-speaking athletes in other countries, translation of the SCAT6 is very important for Turkish healthcare professionals. In addition, the translation of SCAT6 into Turkish is also important in terms of collecting normative data from Turkish-speaking athletes The aim of this study is to translate the SCAT6 and its instructions into Turkish and to carry out its cross-cultural adaptation. This tool is aligned with the SKDA6™ tool published by Concussion in Sport Group – British Journal of Sports Medicine (CISG-BJM).

## Methods

**Fig. 1:**
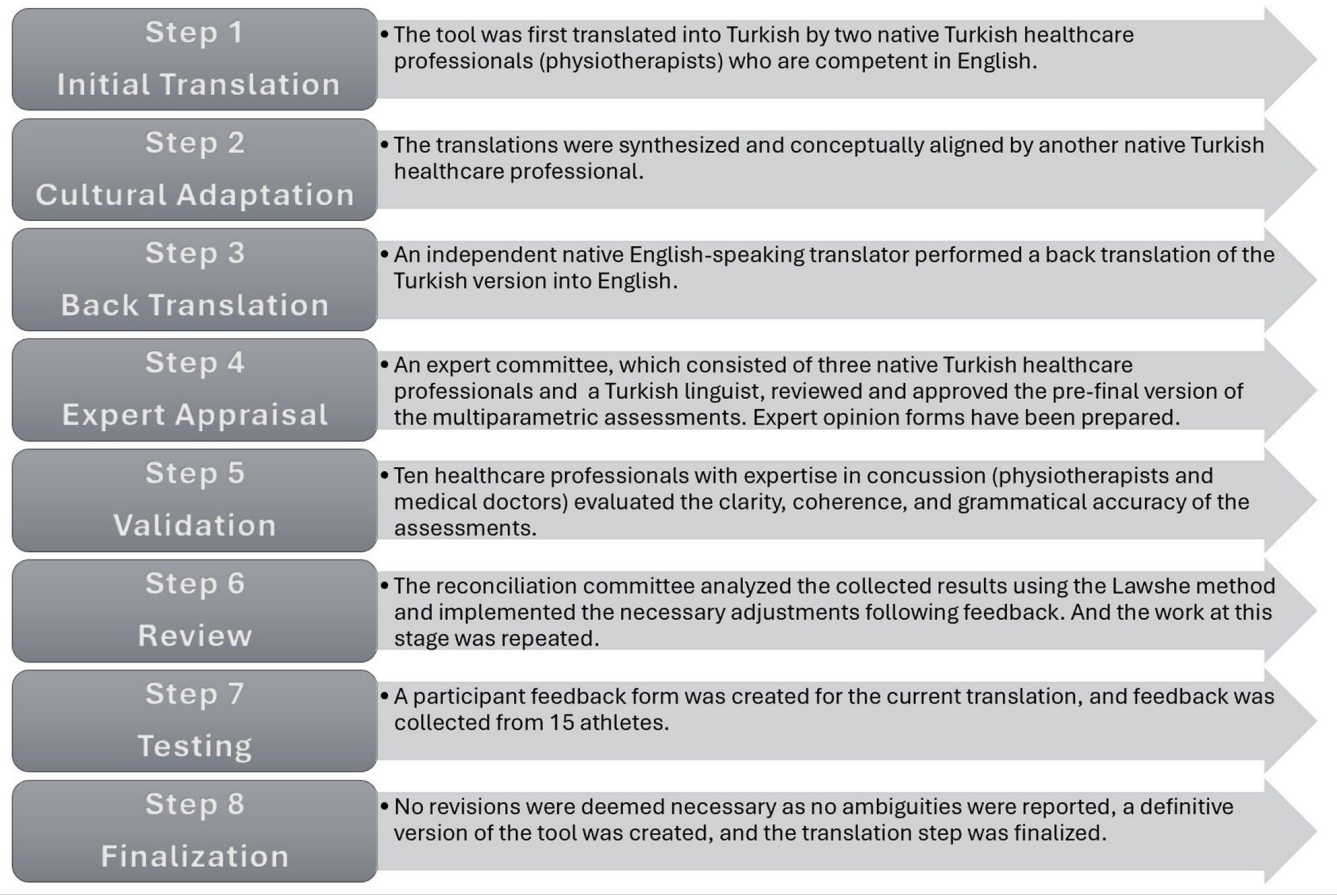
The workflow diagram for translation and adaptation process

First, we obtained permission by filling out a detailed proposal form related to the work plan from the CISG-BJM Tools Committee which prioritises standardising all translation studies of SCAT. Then, another consent was obtained from the Non-Interventional Ethics Committee of Ordu University (number:………….), and the entire study process was conducted in accordance with the Declaration of Helsinki. The Turkish translation and cross-cultural adaptation of the SCAT6 process was carried out in 8 stages according to international guidelines [13, 14]. The workflow diagram is shown in Fig. 1. Once the final version of the form had been agreed upon, validity and reliability analyses were performed.

### Questionnaires

#### Sport Concussion Assessment Tool 6 (SCAT6)

The original form has been translated into Turkish and used as such. As explained in the introduction to SCAT6, it consists of two main sections: Immediate Assessment /Neuro Screen and Off-Field Assessment. The Immediate Assessment / Neuro Screen section begins with a red flag diagram and comprises five steps: Observable Signs, Glasgow Coma Scale, Cervical Spine Assessment, Coordination & Ocular/Motor Screen, and Memory Assessment with Maddocks Questions. Off-Field Assessment consists of six steps: Athlete Background, Symptom Evaluation, Cognitive Screening, Coordination, and Balance Examination, Delayed Recall, and a Decision Checklist. Some parts generate quantitative performance scores, whereas the others are interpreted as dichotomous variables. (normal/abnormal). Thus, it does not have a total diagnostic score. Symptom scores do not provide diagnostic certainty, but they do offer sensitive indicators of clinical status [6]. 

#### The Oslo Sports Trauma Research Centre Questionnaire on Health Problems (OSTRC-H)

OSTRC-H is a self-report measurement tool developed by the Oslo Sports Trauma Research Centre to systematically and sensitively monitor both injury- and illness-based health problems in athletes. It consists of four core questions that assess the athlete’s level of restriction in training and competition participation, performance impairment, symptom severity, and general health-related problems over the past 7 days. The scale contains 4 questions with options ranging from 0-8-17-25. The total score is between 0 and 100 points. Higher points indicate worse health conditions [15].

OSTRC-H is widely used in epidemiological follow-up studies, research investigating the relationship between workload and injury, and weekly monitoring of health status changes in athletes due to its high sensitivity and applicability. A Turkish version is available [16].

#### Translation and cross-cultural adaptation process

##### Step 1: initial translation

At this stage, to ensure proficiency in content and terminology, it was decided that the translators should be healthcare professionals. The form was translated into Turkish by two independent translators whose native language is Turkish and who also possess advanced English skills. 

##### Step 2: integration of translations

The translated drafts were synthesized and conceptually aligned by another native Turkish healthcare professional. 

##### Step 3: back-translation

The Turkish version was back translated into English by a bilingual translator who is blind to the original version. 

##### Step 4: expert appraisal

The texts (original, translated, and back-translated versions) were reviewed in terms of clinical coherence and semantic features by a working committee of experts in this field, comprising three physical therapists, a medical doctor, a sports scientist, and a linguist. For a systematic screening, the form sections were examined in detail under 21 subheadings. For a functional equivalence, the words in the Immediate Memory subtest were replaced with words that are at the same level in terms of developmental and linguistic difficulty and have similar frequencies of use in Turkish. Words were selected from a Turkish word list established in the literature, following the recommendations of linguists. Since the names of the months in Turkish are different from those in English, no changes were made for the Months in Reverse Order Test. An Expert Opinion Form was created for the revised form and sent to experts not on the working committee. 

##### Step 5: validation

The experts were asked to review the form and evaluate the appropriateness of the section translations according to a 3-point Likert scale (appropriate, partially appropriate, not appropriate) for the 21 subsections, and to provide recommendations for parts they thought needed to be changed or improved. 

##### Step 6: review

The results obtained from 10 experts were analyzed using the Lawshe Method, and the opinions were evaluated by the committee. Changes agreed upon were implemented and reshared with the experts for reassessment. It was determined that the excellent Content Validity Ratio (CVR) had been achieved by the second round, so a pre-final version was approved for testing. 

##### Step 7: testing of the prefinal version

A Likert type “Participant Feedback Form” was created to evaluate the clarity and comprehensibility of the translated form. Feedback was collected from 15 athletes. 

##### Step 8: finalization

No revisions were deemed necessary as no ambiguities were reported. A definitive Turkish version of the SKDA6™ was created, and the translation step was finalized.

### Validity and reliability process

Feedback from experts and participants also examines content validity. The Turkish version of OSTRC-H scale was used to assess the concurrent validity of the Symptom Severity section.

The reliability of the form was tested using interclass correlation and intraclass correlation methods. Athletes were evaluated by different assessors 6.94 ± 0.93 days apart to reduce the risk of recalling answers (Test 1 by Tester A - Test 2 by Tester B). The first assessor re-evaluated the athletes 14.04 ± 1.68 days after the initial measurement (Test 3 by Tester A).

Because the Immediate Assessment/Neuro Screen section primarily consists of observational or dichotomous clinical judgments rather than quantitative outcomes, reliability analyses were prospectively restricted to quantitative Off-Field Assessment components (Steps 2–5). Because SCAT6 is not a unidimensional scale and does not yield a total score, reliability analyses were conducted separately for each quantitative subsection rather than for the instrument as a whole.

### Subjects

**Fig. 2:**
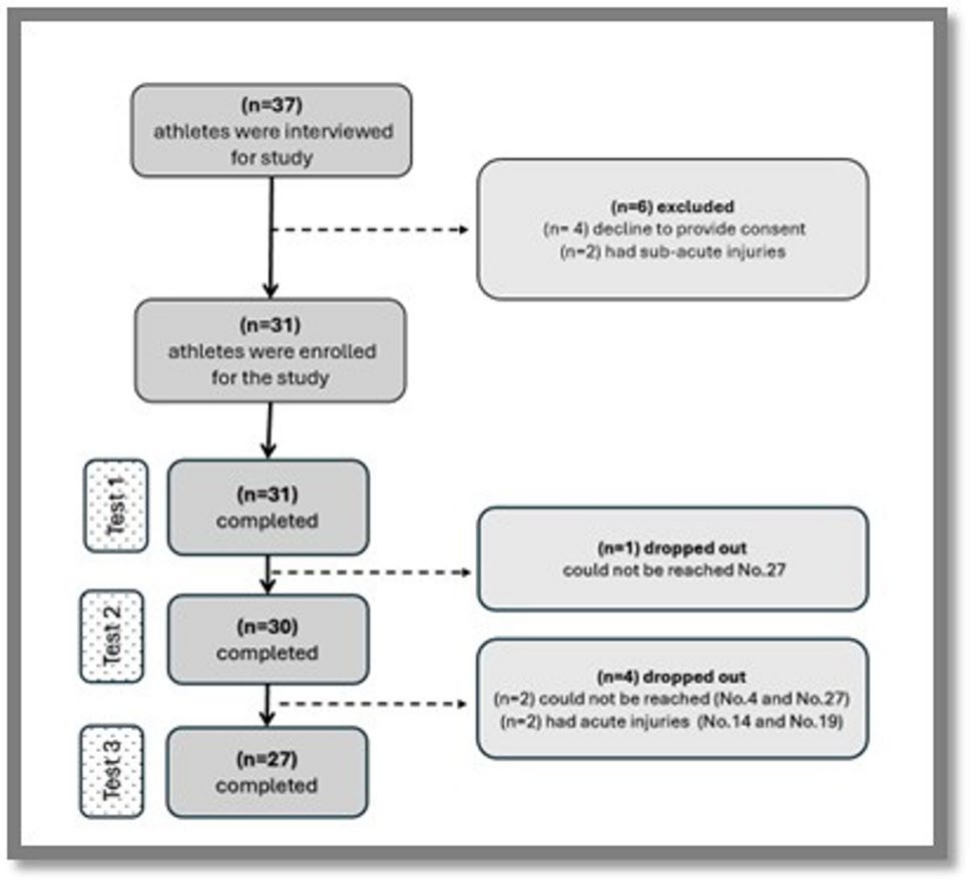
Participants flow chart

All athletes over 16 years of age who were literate and able to speak Turkish fluently were included in the study. Athletes who sustained a head injury or lower limb injury, or who experienced clinical changes, during the test-retest interval (within two weeks of the initial assessment) were excluded from the study. For the longitudinal assessment, all participants were identified by number (No.1–31). Thirty-seven athletes were contacted for the study, and 31 athletes who agreed to participate in the study were evaluated. The work was completed with 27 people due to injuries during the process and a communication breakdown. The number of participants included in the different evaluation stages is shown in Fig. 2.

### Statistical analysis

The content validity of SKDA6™ was assessed using the Lawshe method, which quantitatively measures expert consensus on the appropriateness of items [17]. The Lawshe method calculations were performed by using Excel-Microsoft 365 (Version 2501, Build Microsoft Corporation), and other statistical analyses were performed with the Statistical Package for Social Sciences Program (version 22.0 SPSS for Windows, Inc, Chicago, IL). In this approach, the SKDA6™ assessment form was divided into 21 subheadings and submitted for expert review. Each expert evaluated whether a section was “appropriate,” “partially appropriate,” or “inappropriate.” Then, CVR is calculated for each item based on the percentage of experts who rated it as appropriate. CVR values are compared with Lawshe’s critical threshold values to determine whether an item has sufficient content validity. Finally, the Content Validity Index (CVI) was calculated to provide an overall measure of the scale’s content validity [17–19].

“Symptom Evaluation” subsection, which examines the symptoms of concussion injuries used in the assessment, is considered to be related to the overall assessment. Thus, the relationship between the scores collected was evaluated using Spearman’s correlation analysis method to investigate concurrent validity [20].

ICC values were used to assess inter-rater reliability, intra-rater reliability, and supplementary overall three-measurement reliability [21, 22]. Pairwise inter-rater reliability was evaluated between Test 1 and Test 2 using ICC[2, 1], whereas pairwise intra-rater reliability was evaluated between Test 1 and Test 3 using ICC[3, 1]. In addition, supplementary overall reliability analyses incorporating all three available measurements (Test 1 by Rater A, Test 2 by Rater B, and Test 3 by Rater A) were conducted for each quantitative subsection using a two-way mixed-effects model with absolute agreement and single-measure ICCs.

Initially, the sample size was calculated for the overall three-measure ICC analysis, in which Test 1, Test 2, and Test 3 were considered simultaneously, according to the ICC-based reliability framework proposed by Walter et al. [23]. The calculation was based on a one-sided hypothesis framework testing whether the expected ICC exceeded the minimum acceptable ICC. The minimum acceptable ICC was set at ρ₀ = 0.60 and the expected ICC at ρ₁ = 0.80, with an alpha level of 0.05, 80% power, and three measurements per participant. Under these assumptions, the required minimum sample size was 22 participants. To allow for potential attrition, 37 athletes were contacted, and 31 eligible athletes participated in the first assessment. Due to dropouts, the analyzed data were available from 31 participants for concurrency analysis (Test 1), 30 participants for the inter-rater analysis (Tests 1,2), and 27 participants for the intra-rater analysis (Test 1,3) and the overall three-measure ICC analysis (Tests 1,2,3). Although the sample size was initially calculated based on the three-measure ICC analysis, as mentioned above, the primary reliability analyses focused on the inter-rater and intra-rater reliability values, which are two-measure ICC analysis. We acknowledge that the current sample size may have reduced the statistical sensitivity of the two-measure ICC analyses and widened the confidence intervals. This issue has therefore been considered a limitation of the study. ICC values were interpreted as follows: poor (ICC < 0.50), moderate (0.50 ≤ ICC < 0.75), good (0.75 ≤ ICC < 0.90), and excellent (ICC ≥ 0.90) [24].

The validity of the form was assessed using test-retest methods. The consistency of the results obtained by different evaluators for the same athletes was investigated for the ICC value. The consistency of the results obtained by the same evaluator at various times was also evaluated for the ICC value [21, 22, 24].

## Results

Demographics and outcomes of participants are included in Table 1. In this study, 31 participants were included in the initial assessment, and 27 participants remained in the final retest (Fig. 2).

**Table 1:** Demographics of the athletes

Total participants *n*		TEST 1	TEST 2	TEST 3
		31	30	27
SEX n (%)	Female	11(35.5)	10(33.3)	10(37.03)
	Male	20(64.5)	20(66.6)	17(62.97)
Age (years) (Mean ± SD)		20.93 ± 3.98	21.16 ± 3.83	21.03 ± 4.00
Sport duration (%)		9.25 ± 4.53	9.46 ± 4.45	9.62 ± 4.46
Sport type n (%)	Taekwondo	12(38.7)	11(36.7)	11(40.7)
	Football	10(32.3)	10(33.3)	8(29.6)
	Handball	8(25.8)	8(26.7)	8(29.6)
	Boxing	1(3.2)	1(3.3)	0
Previous Concussion n (%)	Yes	8(25.8)	8(26.6)	6(22.2)
	No	23(74.2)	22(73.3)	21(77.8)

*SD* Standard Deviation

### Content validity

The Minimum Acceptable CVR was calculated to be 0.62 for the evaluation of the Expert Opinion Form, conducted with 10 participants. And the CVR value was determined for each section. It was observed that 6 out of 21 sections were below the threshold value **(**Table 2**)**. The working committee made the necessary changes, taking into account the feedback from the experts. The new version of the form was sent back to the same experts for evaluation. In the second round, all CVR values exceeded the threshold value and reached a high degree (0.80-1). The CVI value was calculated to be 0.90 for the SKDA6™.

**Table 2:** Content validity analysis by lawshe method

Section	Subsection	Tests	Parts	CVR-1	CVR-2
Explanation and Information Section	What is SCAT6?		1	0.2*	0.8
	Identification and Transfer		2	0.8	0.8
	Key Points		3	0.4*	1
	Reminders		4	0.8	1
Emergency Assessment / Neuro Screening (On-Field Assessment)	Step 1: Observable Signs		5	0.4*	0.8
	Step 2: Glasgow Coma Scale		6	0.8	1
	Box 1. Red Flags		7	0.8	0.8
	Flags		8	1	1
	Step 3: Cervical Spine Assessment		9	0.8	0.8
	Step 4: Coordination and Ocular/Motor Screen		10	1	1
	Step 5: Memory Assessment		11	1	1
Off-Field Assessment	Maddocks Questions		12	0.6*	0.8
	Step 1: Athlete Background		13	0.4*	0.8
	Step 2: Symptom Evaluation		14	1	1
	Step 3: Cognitive Screening	Orientation	15	0.8	1
		Immediate Memory	16	0.8	0.8
		Concentration	17	0.4*	1
	Step 4: Coordination and Balance Examination	mBESS	18	0.8	1
		Timed Tandem Gait	19	0.8	0.8
	Step 5: Delayed Recall		20	1	1
	Step 6: Decision		21	0.8	0.8

*CVR* Content Validity Ratio, *SCAT* Sport Concussion Assessment Tool, *mBESS *Modified Balance Error Scoring System Test

*Below the minimum acceptable CVR (0.62)

### Concurrent validity

Correlation of OSTRC-H and the “Symptom Evaluation” part in step 2 was evaluated with the Spearman correlation coefficient. Moderate positive correlations were found between OSTRC-H and both the total number of symptoms and symptom severity score (*p* < 0.01) **(**Table 3**)**.

**Table 3:** Correlation values of symptom evolution and OSTRC-H questionnaire

SYMPTOM EVOLUTION (*n* = 31)	OSTRC-H (*n* = 31) mean ± SD (21.51 ± 25.51)	
	*r*	*p*
Total number of symptoms mean ± SD (6.77 ± 4.34)	0.607	< 0.001
Symptom severity score mean ± SD (14.51 ± 12.20)	0.666	< 0.001

*SD* Standard Deviation, *OSTRC-H* The Oslo Sports Trauma Research Centre Questionnaire on Health Problems

### Reliability analyses

Inter-rater and intra-rater reliability estimates for quantitative off-field assessment components are presented in Table 4. Symptom evaluation showed good-to-excellent reliability, whereas cognitive screening, coordination and balance examination, and delayed recall demonstrated moderate-to-good reliability across assessments. Supplementary overall three-measurement ICC analyses demonstrated moderate-to-good reproducibility across quantitative SKDA6™ subsections. Symptom evaluation subsections showed good overall reliability, whereas cognitive and balance-related subsections demonstrated moderate-to-good overall reliability (Table 4).

**Table 4:** Outcome Scores of SKDA6™ and Results of Reliability Analysis

	Outcome Scores (Mean ± SD)			Reliability		
	Test 1 *n* = 31	Test 2 *n* = 30	Test 3 *n* = 27	Inter-rater ICC T1-T2	Intra-rater ICC T1-T3	Overall 3-measurement ICC (95% CI) T1-T2-T3
Step 2 Symptom Evaluation						
Total number of symptoms	6.77 ± 4.34	5.30 ± 4.30	5.25 ± 4.33	0.902***	0.820**	0.782** (0.62–0.88)
Symptom severity score (points)	14.51 ± 12.20	9.86 ± 10.17	9.85 ± 9.92	0.906***	0.831**	0.801** (0.61–0.90)
Step 3 Cognitive Screening						
Immediate memory (points)	20.29 ± 2.00	20.50 ± 3.12	21.22 ± 2.20	0.623*	0.722*	0.532* (0.35–0.72)
Concentration MIROT+DWBT (seconds)	3.67 ± 0.97	3.86 ± 1.22	3.77 ± 0.93	0.661*	0.688*	0.645* (0.44–0.80)
Total Cognitive Point	35.38 ± 2.98	35.23 ± 4.64	36.14 ± 3.31	0.653*	0.676*	0.684* (0.49–0.83)
Step 4 Coordination and Balance Examination						
mBESS	2.19 ± 1.49	1.86 ± 1.27	2.29 ± 1.20	0.735*	0.802**	0.642* (0.43–0.79)
Timed Tandem Gait (seconds)	10.68 ± 2.75	9.74 ± 2.20	9.78 ± 2.17	0.814**	0.735*	0.764** (0.51–0.89)
Dual Task Gait^†^ (seconds)	25 ± 8.77	23.79 ± 7.61	23.63 ± 7.20	0.752**	0.839**	0.700* (0.48–0.85)
Step 5 Delayed Recall	6.38 ± 1.52	5.93 ± 1.81	5.96 ± 1.22	0.680*	0.825**	0.624* (0.45–0.78)
Total Test Duration (seconds)	13.53 ± 1.36	13.40 ± 1.35	12.79 ± 1.21			

*SD* Standard Deviation, *CI* Confidence Interval, *MIROT* Months in Reverse Order Test, *DBWT* Digit Backwards Test, *mBESS *Modified Balance Error Scoring System Test

*Moderate reliability (ICC 0.50–0.75), **Good reliability (ICC 0.75–0.90), ***Excellent reliability (ICC > 0.90) ^†^Error scoring was not considered for the dual-task gait assessment

According to these evaluations;

- The Total number of symptoms and Symptom severity score in the Step 2 Symptom Evaluation section were found to have excellent interrater and good intrarater reliability.
- Interrater and intrarater reliability for all subtests in the Step 3 Cognitive Screening section were found to be moderate.
- The reliability of the tests in the Step 4 Coordination and Balance Examination section was found to be moderate to good.
- Step 5 The delayed recall section was also found to have moderate interrater reliability and good intrarater reliability.

## Discussion

The aim of this study was to establish the translated and cross-cultural adaptation of the Turkish version of SCAT6, which provides the opportunity to assess concussions occurring in sport using a standard form. SKDA has been translated into Turkish in accordance with international guidelines, and validity and reliability analyses have been conducted.

The most fundamental change during cross-cultural adaptation was made in relation to the words under the headings of immediate memory and delayed recall. The words included here were determined with the support of a linguist, based on reference articles [25].

The content validity was assessed by 10 different experts (physiotherapists and medical doctors) using the Lawshe method for each of the 21 subheadings of the form. The content validity ratio (CVR) was determined to be 0.62. In the initial assessment, some items fell below this ratio. Based on the feedback received, the form was updated and shared with the experts for reassessment. As a result, an average CVR value of 0.90 was achieved. This value indicates high content validity. Similar to the current study, CVR values for the Persian version of SCAT6 in the literature ranged from 0.76 to 0.98. The Italian version used a 7-point Likert scale for validity, and items scoring above 5 were considered valid.

One of the most frequent comments we received during the expert review phase was regarding the Turkish translation of the abbreviation modified Balance Error Scoring System (mBESS), and this has been corrected. We received feedback regarding the understanding of the application in certain sections of the form (particularly the immediate memory, concentration, and mBESS sections). Following this feedback, we did not make any changes to the original form, but we created a separate detailed instruction form for the practitioner.

SCAT6 states that the form cannot be administered correctly in less than 10–15 min. In line with these values, the average administration time for SKDA6™ was found to be 13.53 ± 1.36 min.

The current study demonstrated that SKDA6™ possesses inter-rater and intra-rater reliability for assessments of immediate memory, concentration, mBESS, timed tandem gait, dual task gait, delayed recall, and total cognitive scores. The Korean version of SCAT6 also exhibits similar reliability values ( ICC for Immediate memory: 0.816, ICC for tandem gait speed: 0.853, ICC for dual task gait: 0.887,ICC for delayed recall: 0.741,ICC for balance error 0.691) (9). However, in the Korean version, assessments were conducted twice on the same day by the same evaluator. In the present study, the second assessment was performed by another healthcare professional one week later. The third measurement was performed by the same healthcare professional two weeks after the first assessment. Thus, inter-rater and intra-rater reliability tests were performed with an adequate test-retest interval. Supplementary overall ICC analyses incorporating all three assessment occasions also demonstrated moderate-to-good reproducibility across quantitative subsections. Slightly lower overall ICC values observed in some cognitive subsections may reflect expected temporal variability and potential learning effects associated with repeated cognitive assessments.

On the other hand, the Persian version of SCAT6 did not include a reliability test, and it was argued that it was not reasonable to perform this test on healthy athletes [10]. However, it should not be forgotten that this assessment tool can be used to collect data not only from athletes who have suffered concussions but also from healthy athletes. Therefore, it is important to demonstrate the reliability of these tests.

Correlation with the Turkish version of OSTRC-H was examined to test the validity of the symptom evaluation section of SKDA6™, which is completed by the athlete a moderate level of significant correlation was found between the two questionnaires. The OSTRC-H focuses on health problems experienced by athletes in the last 7 days and the effects of these problems [15, 16]. The symptom evaluation included in the SKDA6™ assesses the subjective severity of concussion-related symptoms at the time of evaluation. Although these two questionnaires do not serve the same purpose, both assess symptoms. Therefore, a moderate level of correlation is considered methodologically acceptable. Furthermore, the reliability test for the symptom assessment section of the SKDA6™ was conducted with assessments performed one week apart and found to be reliable. After the items under this heading were translated, they were sent to athletes for comprehensibility and were finalised based on their feedback.

Although the achieved sample size was adequate for the supplementary three-measurement ICC analyses according to the predefined reliability assumptions, inter-rater and intra-rater ICC analyses may still be considered relatively limited in terms of precision. Therefore, future studies with larger samples may further improve the stability and precision of ICC estimates.

Overall, the observed reliability coefficients support the reproducibility of SKDA6™ across symptom, cognitive and balance-related domains.

## Conclusion

The observed ICC values (0.62–0.91 between subscales) fall within the expected range for SCAT related cognitive, balance, and symptom-based assessments. The findings of this study demonstrate that SKDA6™ can be considered a valid and reliable instrument for concussion assessment in Turkish-speaking athletic populations. In addition to its use as a tool to assist with concussion diagnosis, SKDA6™ is an essential contribution to the Turkish literature on sports medicine and offers a framework for healthcare professionals to facilitate recovery and safe return to play. Finally, with this instrument available to researchers and clinicians, it is hoped that normative data can be established for Turkish athletes of all levels, thereby improving the accuracy of concussion management strategies.

## Supplementary Information

Supplementary Material 1.

Supplementary Material 2.

## Data Availability

The datasets used and analysed during the current study are available from the corresponding author on reasonable request.

## Abbreviations

BESS /mBESS: Balance Error Scoring System / Modified Balance Erros Scoring System
CISG: Concussion in Sport Group
CVI: Content Validity Index
CVR: Content Validity Ratio
DBWT: Digit Backwards Test
ICC: Intraclass Correlation Coefficient
MIROT: Months in Reverse Order Test
mTBI: Mild Traumatic Brain Injury
OSTRC-H: Oslo Sports Trauma Research Centre Questionnaire on Health Problems
SAC: Standardized Assessment of Concussion
SCAT6: Sport Concussion Assessment Tool 6th Edition
SKDA6™: Sport Concussion Assessment Tool 6th Edition Turkish Version
SPSS: Statistical Package for Social Sciences
SRC: Sport-Related Concussion
