# Supplementary material for: Turkish translation and cross-cultural adaptation of the Sport Concussion Assessment Tool 6th edition (SCAT6)
Source: BMC Sports Sci Med Rehabil. 2026 Jun 3;18:348. doi: 10.1186/s13102-026-01784-9 (PMC13455324; doi:10.1186/s13102-026-01784-9)
Supplement: Supplementary file 1 [file 13102_2026_1784_MOESM1_ESM.docx]

**Translation/Adaptation Proposal: CISG SCAT6 Tools**

**Proposal title**

Turkish translation and cross-cultural adaptation of the Sport Concussion Assessment Tool 6 (SCAT6)

**Investigators**

1. Yuksel Fuat, Assistant Professor, Ordu University/Faculty of Health Sciences, /Department of Physiotherapy and Rehabilitation, Role: Proposing the study design, Project Coordinator, drafting the manuscript, (consider attaching a short bio)

2.Aygun Polat Elif, Research Assistant, PhD, Ordu University/Faculty of Health Sciences, /Department of Physiotherapy and Rehabilitation, Role: data collection, drafting the manuscript,

3. Yilmaz Ozal Seyda Proposing the study design, statistical analysis, drafting the manuscript,

**Background and rationale (Short explanation as to why the adaptation is warranted)**

Concussions are a significant public health issue, particularly within the realm of sports, where athletes are at an elevated risk of head injuries. Scat 6 is a valid and reliable method for the assessment of athletes who have suffered a concussion both on and off the sidelines. Unfortunately, no SCAT version developed to date has been adapted into Turkish. The translation and cultural adaptation of SCAT6 into Turkish will fill a critical gap in the clinical tools available to Turkish sports medicine professionals. It will allow for more accurate concussion assessments and promote safer management of concussions in Turkish athletes. Additionally, the adaptation will enable future research on concussion in Turkish sports settings, contributing to the global body of knowledge on sports-related head injuries.

**Objectives**

**Primary objective:** To translate and culturally adapt the Sport Concussion Assessment Tool 6 (SCAT6) into Turkish, ensuring linguistic accuracy and cultural relevance for use in Turkish sports settings.

**Secondary objective:** To ensure the correct follow-up of concussion injuries in Turkish-speaking athletes.

**Research design**

**Research methodology**

The processes of translation, adaptation and translation verification will be based on prior

research.

Step 1: Initial Translation

At this stage, two independent bilingual translators whose native language is Turkish will translate the content into Turkish.

**Step 2: Integration of Translations**

A single translation will be created by comparing the two Turkish versions of SCAT 6.

**Step 3: Back-translation**

The Turkish version will be translated into English by 2 bilingual colleagues who are blind to the original version. The Turkish version will be translated into English by 2 bilingual colleagues who are blind to the original version. These two English versions will be compared, differences will be resolved by consensus and a new combined version will be created. If consensus cannot be reached, a third person will be asked for help.

**Step 4: Expert Evaluation**

The texts (translate and back translate) will be reviewed by a committee of experts in the field (physiotherapists, orthopaedic physicians, sports scientists, linguists) and compared with the original text. Suggestions will be evaluated, and a prefinal version will be created for testing.

**Step 5: Test of the Prefinal version**

The text created in Turkish will be tested on twelve professional athletes. Comments will be collected in terms of clarity, intelligibility, and consistency.

**Step 6: Finalization**

The comments of the athletes will be evaluated by the researchers and necessary changes will be applied to the text. The final version of the Turkish version of SCAT 6 will be created.

**Test-retest reliability**

**Target Population**

1. Inclusion Criteria

2. Exclusion Criteria

**Data and statistical analysis**

**1.** Test-retest reliability

**Proposed Timeline**

| **Method** | **Oct 24-Dec 24** | **Jan-Mar 25** | **April- June25** | **July- Oct24** | **Nov-Dec24** |
| --- | --- | --- | --- | --- | --- |
| **1. Set the topic, review literature and**  **writing research proposal** |  |  |  |  |  |
| **2. Approval of the ethics committee** |  |  |  |  |  |
| **3. Translation process and data**  **collection** |  |  |  |  |  |
| **4. Data analysis and conclusion** |  |  |  |  |  |
| **5. Publication process** |  |  |  |  |  |
